# The relationship between community food environment around schools and student meal participation: the role of school CEP participation status

**DOI:** 10.1186/s12916-024-03498-6

**Published:** 2024-07-09

**Authors:** Emily M. Melnick, Francesco Acciai, Michael J. Yedidia, Punam Ohri-Vachaspati

**Affiliations:** 1 College of Health Solutions, Arizona State University, Phoenix, AZ USA; 2 Rutgers Center for State Health Policy, Rutgers University, New Brunswick, NJ USA

**Keywords:** School meal participation, Food environment, Community eligibility provision

## Abstract

**Background:**

Despite the many benefits of school meals, not all students participate. One reason students may not participate in school meals is because they instead purchase breakfast or lunch from food outlets located around schools that mostly carry unhealthy items. This study examined whether school participation in the Community Eligibility Provision (CEP), which allows qualifying schools to serve free meals to all students, moderated the association between the community food environment around schools and student meal participation.

**Methods:**

This study employed a longitudinal repeated-measures design using school-level data collected between 2014 and 2020 within four low-income school districts (*n* = 126 schools) in the US. We obtained meal participation data from state records and created a measure characterizing the community food environment within 0.25 miles of schools (characterized as low-density of unhealthy food outlets vs. high-density of unhealthy food outlets) through a latent class analysis. Regression analysis estimated associations between community food environments, CEP participation, and participation rates in school breakfast and school lunch, assessed in separate models.

**Results:**

While no moderating effect of school CEP status was observed for breakfast or lunch participation, school breakfast participation was predicted to be 4% lower in high-density food environments than in low-density environments (*P*-value = .049) among non-CEP schools, and there was no difference in participation by the community food environment among CEP-participating schools. Differences in breakfast participation by the community food environment among non-CEP schools were mostly attributable to middle/high schools, with participation predicted to be 10% lower in high-density environments than in low-density environments among non-CEP middle/high schools (*P*-value < .001), whereas such a difference in participation was not observed among non-CEP elementary schools.

**Conclusions:**

Negative associations between food environment around schools and school breakfast participation were observed only among middle and high schools not participating in CEP, suggesting that policy actions to increase access to free school meals may benefit students, particularly older children and adolescents.

**Supplementary Information:**

The online version contains supplementary material available at 10.1186/s12916-024-03498-6.

## Background

School meals provide important nutritional support for school-aged children and adolescents in the US, particularly for students living in families at increased risk of experiencing food and nutrition insecurity [1, 2]. Demonstrated benefits of school meals include improved nutrition, academic, and behavioral outcomes [3, 4]. These benefits have been particularly pronounced since the passage of the Healthy Hunger-Free Kids Act of 2010, which requires that school breakfasts and lunches meet healthier nutrition standards [5]. Studies conducted following the passage of this legislation have shown that both lunches and breakfasts consumed at school are more nutritious than meals brought from home or purchased in other places [6–9]. However, despite the many benefits of school meals, not all students participate. A few possible reasons for non-participation include perceived stigma related to participating in school meals, bringing food from home instead, and lack of availability of preferred food items [10]. Another reason students may not participate in school meals is because they instead purchase breakfast or lunch from food outlets, such as fast-food restaurants and convenience stores, which are often located around schools [11–13]. Research conducted in the US and other countries indicates that a higher density of fast-food outlets surrounding schools is associated with lower participation in school meal programs [14, 15] and the foods that students tend to purchase from food outlets surrounding schools (including fast-food restaurants and convenience stores) are often of poorer nutritional quality compared to school meals [16, 17]. Given the benefits of school meals and the differences in nutritional quality between school meals and food purchased in retail outlets in the surrounding community, it is important to consider how school policies and programs may boost school meal participation rates.

The Community Eligibility Provision (CEP) authorized by the Healthy Hunger-Free Kids Act of 2010 allows US schools and districts serving children from low-income areas to offer meals at no cost to all enrolled students [18]. The aim of CEP is to improve access to nutritious school meals among children living in low-income areas. Schools are eligible to participate in CEP based on the proportion of students whose families participate in other means-tested federal assistance programs; this proportion is known as the identified student percentage. Schools were eligible to participate in CEP if they had an identified student percentage of 40% or higher until school year (SY) 2022–2023 [18]. Beginning October 26, 2023, the US Department of Agriculture expanded access to CEP by lowering the identified student percentage requirement to 25% or greater [19]. Notably, not all schools or districts that are eligible to participate in CEP choose to do so. For some schools, participating is not financially viable due to the reimbursement structure, which is dependent on the school’s identified student percentage [20]. Additional concerns expressed by eligible schools opting not to participate in CEP include a lack of clarity about the implementation of CEP, anticipated operational challenges associated with implementing the program, and uncertainty regarding the impacts of CEP on school meal participation [21]. In SY 2019–2020, only 69% of eligible schools in the US opted to participate in CEP [22].

Schools participating in CEP tend to have higher rates of school meal participation, compared to similar, eligible schools that do not participate in CEP, in part due to a normalization of eating school meals and reduction of associated stigma [3, 20, 23]. Consequently, one may hypothesize that school participation in CEP may lessen the attraction of unhealthy food outlets surrounding schools for students, thereby providing a “buffering effect” against obesogenic community food environments that are often associated with reduced student participation in school meals. It is also possible that older students are more susceptible to influences of community food environments surrounding schools, since they may have more purchasing power to pick up foods from nearby outlets [13] and are more likely to travel to school unsupervised by a parent [24]. To test these hypotheses, we investigated whether the association between the community food environment (specifically, the density of unhealthy retail food outlets) around schools and school meal participation rates differed based on schools’ CEP participation status, using longitudinal data collected over a 6-year period within four urban public school districts in the US. Anticipating age-related differences in consumption behaviors, we also investigated whether this association was different for elementary vs. middle and high schools.

## Methods

This study employed a longitudinal repeated-measures design to evaluate associations of interest using data collected from public schools between SY 2014–2015, when CEP became available to eligible schools nationwide in the US, and SY 2020–2021. Data were collected from all public schools within four urban school districts in New Jersey (Camden, New Brunswick, Newark, and Trenton) with predominantly low-income, high-minority populations. There were 126 public schools with complete data on all collected variables across the four school districts over the study period. There were 98 schools with complete data in SY 2014–2015, 98 in SY 2015–2016, 99 in SY 2016–2017, 101 in SY 2017–2018, 84 in SY 2018–2019, and 91 in SY 2019–2020 (total *n* = 571 observations). In addition to non-response, which was limited, the number of schools for each SY differed slightly from year to year due to school openings and closings. The majority of schools in the sample (69.4%) were observed in all six study years. All schools within the study sample were low-income; 82.6% of students attending schools not participating in CEP and 67.3% of students attending CEP-participating schools were eligible for free and reduced-price meals over the study period. Additionally, in 2019–2020, all schools within the study sample were eligible to participate in CEP based on identified student percentage.

### Datasets

Longitudinal data were collected between SY 2014–15 and SY 2019–20 as part of the New Jersey Child Health Study and were used to characterize the community food environment around schools and the food environment within schools [25]. Measures of the community food environment around schools were based on lists of food outlets obtained from two commercial data companies, InfoUSA [26] and Trade Dimensions/Nielsen [27], for each study year within the cities where the four school districts were located. Comprehensive school data were collected through a 96-item survey, including questions based on previous research [28, 29], that was completed by school nurses, in consultation with school food staff. The survey could be completed through an online survey platform (Qualtrics – Provo, UT, USA) or with pen and paper. To determine school breakfast and lunch participation rates for each school for each of the SYs, we obtained school-level average daily participation numbers in school breakfast and lunch and school enrollment data from the New Jersey (NJ) Department of Agriculture through Open Public Records Act requests. For SY 2019–2020, average daily participation numbers in school lunch and school breakfast programs were calculated based on participation between the start of the school year and March 2020, when schools were closed due to the COVID-19 pandemic. The NJ Department of Education and Food Research & Action Center websites provided data on school participation in CEP for each of the SYs [30, 31]. Finally, data from the National Center for Education Statistics common core data repository provided school-level characteristics, including the proportion of students eligible for free or reduced-price meals (FRPM) and the racial/ethnic composition of enrolled students, for each of the SYs over the study period [32].

### Outcome variables

The outcome variables for all analyses were the (1) school breakfast participation rate and (2) school lunch participation rate. Participation rates were calculated for each of the SYs under investigation by dividing the average daily participation numbers in school breakfast and school lunch by the number of students enrolled.

### Predictor variables

To characterize the community food environment around schools, we created a measure of *unhealthy food outlet density*. For each study year, lists of food outlets obtained from commercial databases were de-duplicated, geocoded, and categorized into outlet types based on the types of foods served and sales volume using a systematic classification protocol [33]. This study included two types of food outlets that are both likely to be frequented by students and carry a majority of unhealthy items: convenience stores and limited-service (i.e., fast-food) restaurants. All stores with a sales volume less than $1 million and stores from larger chains recognized as convenience stores (e.g., Wawa or 7-Eleven) were classified as convenience stores. Additionally, stores with sales volume between $1 and $2 million were classified as convenience stores if they did not carry 3 or more of the following healthy options: 5 different types of fruits, 5 different types of vegetables, low-fat or skim milk, and fresh or frozen meat. Restaurants in which patrons paid before, rather than after, eating were classified as limited-service restaurants.

To determine the prevalence of these two types of food outlets around schools, we calculated the number of outlets within a 0.25-mile roadway network—a walking distance consistent with previous literature [34, 35]—from each school. Distances were calculated using the most direct route by means of roadway networks obtained using NJ Road Network data from the NJ Department of Transportation and NJ Office of GIS [36], excluding highways and toll roads as walking routes, using Geographical Information System ArcGIS software (Esri, Redlands, CA).

Latent class analysis models (via generalized structural equation modeling) were used to create the unhealthy food outlet density measure across the assessed 6-year period. Variables used to create this measure included (1) the number of convenience stores and (2) the number of limited-service restaurants. These two variables were upcoded (i.e., values were capped at the 97th percentile of the distribution if they were above this threshold) to reduce the influence of extreme values on class assignments. Likelihood-ratio tests, the Akaike information criterion, and the Bayesian Information Criterion assessed the latent class analysis model specification for 2-, 3-, and 4-class models. A 2-class model (low-density environment vs. high-density environment) was retained based on indicators of goodness of fit and because both the 3- and 4-class models produced some classes that were deemed too small to support regression models with interaction terms. For example, one of the three classes in the 3-class solution only had four CEP-participating schools.

School participation in CEP (yes vs. no) was treated as a dichotomous variable for each of the study years.

### Control variables

A number of school-level variables were included as controls in the analyses. School food environment variables included the healthfulness of school foods served at lunchtime, healthfulness of available competitive foods, and breakfast delivery model (breakfast served in the classroom vs. not) based on school staff responses to New Jersey Child Health Study survey items. Consistent with previous literature [34], we created two summary measures (i.e., scales ranging from 0 to 1) to measure school foods healthfulness based on responses to food availability questions, with higher scores corresponding to healthier environments. The first scale, capturing the healthfulness of school foods served at lunchtime (i.e., the *NSLP healthy scale*), was calculated as the ratio of healthy NSLP items over the total (healthy + unhealthy) number of NSLP items. The second scale, capturing the healthfulness of a la carte foods and foods sold in vending machines (i.e., the *Competitive food healthy scale*), was also calculated as the ratio of healthy items divided by the total number of items (healthy + unhealthy). Additional information on the within-school food environment data and the full list of items captured within the scale measures is available elsewhere [37].

In addition, we included as control variables school majority race/ethnicity grouped into three categories: (1) majority (> 50%) black, (2) majority (> 50%) Hispanic, and (3) majority (> 50%) non-Hispanic White/no majority race/ethnicity; school level grouped into two categories: elementary school and middle/high school; school enrollment operationalized continuously; the proportion of students eligible for FRPM (ranging from 0 to 100); and school year operationalized continuously.

### Statistical analysis

Regression models for panel data assessed associations between all predictor variables of interest and participation rates in school breakfast and lunch, analyzed in separate models. Because school meal participation rates are proportions, thus bounded between zero and one, standard linear models, which assume an unbounded distribution of the outcome variable, may not provide an accurate representation of the associations of interest. Therefore, using regression models for fractional outcome variables is more appropriate for the present study [38]. Specifically, we used the xtgee command in Stata version 16 (StataCorp LLC, College Station, TX) to run a generalized linear regression model for panel data with a binomial distribution, probit link function, and robust standard errors. Interaction terms between the community food environment category (high-density environment/low-density environment) and CEP participation (yes/no) were added in subsequent models to examine whether the association between the community food environment around schools and (1) school breakfast and (2) school lunch participation rates, were moderated by CEP participation. To assess whether these interaction effects differed according to school level, we then used a 3-way interaction term that included (i) the community food environment category, (ii) CEP participation, and (iii) school level (elementary school/middle or high school). We used the margins command in Stata to determine predicted participation rates in school breakfast and school lunch; these rates were based on the coefficients estimated from the models described above and were used to construct the presented figures.

We conducted sensitivity analyses to ensure the robustness of findings. First, because Stata does not allow for a 3-level model or a second clustering variable using the xtgee command, we ran three-level mixed-effects linear regression models, which treated schools as repeated observations (as our main model did) that are also nested within school districts. This was done to address concerns about limited variability in CEP participation within districts. Other models treated the time variable as a series of six dummy variables, one for each SY, instead of a continuous variable. Findings at *P* < 0.05 were considered statistically significant.

## Results

### Latent class analysis

Table 1 depicts the two identified classes and their respective predicted mean numbers of convenience stores and limited-service restaurants. The first identified class (*n* = 487, 85% of the sample), labeled “low-density environment,” was characterized by a relatively lower mean number of convenience stores (1.6 stores) and limited-service restaurants (1.4 restaurants). The second identified class (*n* = 84, 15% of the sample), labeled “high-density environment,” had comparatively more convenience stores and limited-service restaurants.

**Table 1:** Composition of the analytic sample by community food environment within a 0.25-mile roadway network surrounding schools

	Community food environment around school	
	Low-density environment (*n* = 487 school observations)	High-density environment (*n* = 84 school observations)
Convenience stores, mean (SD)	1.6 (1.3)	3.7 (1.3)
Limited-service restaurants, mean (SD)	1.4 (1.3)	7.3 (1.9)

### Descriptive results

Table 2 shows school-level characteristics of the sample for each of the 6 years of the study period, stratified by school CEP participation status. The majority of schools (ranging from 69 to 73% for each study year) were elementary schools. Most of the study schools served a majority of Black or Hispanic students. Over all six SYs observed, 83% of students attending non-CEP schools and 67% of students attending CEP schools qualified for free or reduced-price meals. Over all six SYs observed, over three-quarters of students in CEP schools participated in school lunch (78%) and 59% participated in school breakfast. Of students attending non-CEP schools, 69% participated in school lunch and 57% participated in school breakfast.

**Table 2:** Descriptive characteristics of schools by participation in the Community Eligibility Provision (CEP) for each of the six school years (SYs) of collected data^a^

Sample characteristics												
	**SY 2014–2015** Mean (SD) or %		**SY 2015–2016** Mean (SD) or %		**SY 2016–2017** Mean (SD) or %		**SY 2017–2018** Mean (SD) or %		**SY 2018–2019** Mean (SD) or %		**SY 2019–2020** Mean (SD) or %	
	CEP (*n* = 41)	Non-CEP (*n* = 57)	CEP (*n* = 33)	Non-CEP (*n* = 65)	CEP (*n* = 29)	Non-CEP (*n* = 70)	CEP (*n* = 29)	Non-CEP (*n* = 72)	CEP (*n* = 39)	Non-CEP (*n* = 45)	CEP (*n* = 44)	Non-CEP (*n* = 47)
School breakfast participation rate, %	61.5	58.5	65.7	54.2	60.1	54.0	57.0	56.3	54.4	60.8	55.7	61.0
School lunch participation rate, %	75.7	69.5	79.9	68.9	78.8	67.8	77.3	68.2	69.8	78.0	77.6	71.7
Community food environment around school, %												
Low-density environment	90.2	77.2	93.9	81.5	89.7	82.9	89.7	86.1	92.3	82.2	86.4	83.0
High-density environment	9.8	22.8	6.1	18.5	10.3	17.1	10.3	13.9	7.7	17.8	13.6	17.0
School majority race/ethnicity, %												
Majority Black	41.5	47.4	36.4	53.9	27.6	51.4	31.0	48.6	30.8	53.3	25.0	53.2
Majority Hispanic	56.1	47.4	63.6	42.5	65.5	45.7	69.0	47.2	69.2	37.8	72.7	38.3
Majority Non-Hispanic White/no majority race/ethnicity	2.4	5.3	0.0	4.6	6.9	2.9	0.0	4.2	0.0	8.9	2.3	8.5
School level, %												
Elementary school	61.0	80.7	72.7	67.7	72.4	68.6	72.4	69.4	69.2	73.3	65.9	72.3
Middle or high school	39.0	19.3	27.3	32.2	27.6	31.4	27.6	30.6	30.8	26.7	34.1	27.7
Student enrollment	570 (342)	545 (257)	525 (346)	565 (291)	571 (356)	586 (322)	568 (358)	578 (318)	569 (318)	634 (294)	562 (379)	625 (315)
Competitive food—healthy scale	0.6 (0.3)	0.6 (0.2)	0.6 (0.3)	0.6 (0.2)	0.6 (0.2)	0.7 (0.2)	0.6 (0.2)	0.7 (0.3)	0.6 (0.2)	0.7 (0.2)	0.6 (0.2)	0.7 (0.2)
NSLP—healthy scale	0.7 (0.1)	0.7 (0.1)	0.6 (0.1)	0.7 (0.1)	0.8 (0.1)	0.7 (0.1)	0.8 (0.1)	0.7 (0.1)	0.8 (0.1)	0.8 (0.1)	0.7 (0.1)	0.8 (0.1)
Students eligible for FRPM, %	85.8	85.7	58.2	83.3	61.2	82.4	59.3	84.1	63.1	74.6	70.1	83.6
Breakfast served in classroom, % yes	73.2	79.0	78.8	75.4	75.9	81.4	79.3	81.9	84.6	71.1	81.8	70.2

*FRPM* free or reduced-price meal

^a^Schools were observed up to 6 times spanning between SY 2014–2015 and SY 2019–2020

### Model results

Results of initial multivariable regression models for panel data assessing predictors of school breakfast and school lunch participation rates without interaction terms are presented in Table 3. In these models, the density of unhealthy food outlets around schools was not associated with school breakfast participation rates or with school lunch participation rates. School CEP participation was associated with both higher rates of school breakfast participation (*B* = 0.12, *P* = 0.02) and school lunch participation (*B* = 0.13, *P* < 0.01).

**Table 3:** Results from regression models^a^ assessing predictors of average daily participation rates in school breakfast and school lunch among 126 schools observed over a 6-year period^b^

Predictor variable	Coef	95% CI	*P*-value
**School breakfast**			
Participation in CEP (ref: No)			
Yes	0.12	(0.02, 0.22)	.02
Community food environment around school (ref: low-density)			
High-density	− 0.08	(− 0.18, 0.02)	.11
School year	0.00	(− 0.02, 0.02)	.88
School level (ref: elementary school)			
Middle or high school	− 0.67	(− 0.82, − 0.51)	< .01
School majority race/ethnicity (ref: majority Black)			
Majority Hispanic	0.08	(− 0.04, 0.19)	.19
Majority non-Hispanic white/Mixed	0.14	(0.00, 0.28)	.06
Enrollment (in hundredths)	0.00	(− 0.03, 0.02)	.75
Competitive food healthfulness scale	0.06	(− 0.10, 0.25)	.50
NSLP healthfulness scale	− 0.07	(− 0.39, 0.25)	.67
Proportion of students eligible for FRPM^a^	0.30	(0.09, 0.51)	< .01
Breakfast served in classroom (ref: No)			
Yes	0.21	(0.13, 0.30)	< .01
**School lunch**			
Participation in CEP (ref: No)			
Yes	0.13	(0.7, 0.19)	< .01
Community food environment around school (ref: low-density)			
High-density	− 0.01	(− 0.08, 0.07)	.88
School year	0.02	(0.01, 0.03)	< .01
School level (ref: elementary school)			
Middle or high school	− 0.45	(− 0.60, − 0.29)	< .01
School majority race/ethnicity (ref: majority Black)			
Majority Hispanic	0.10	(0.02, 0.18)	.02
Majority non-Hispanic white/Mixed	0.14	(0.05, 0.24)	< .01
Enrollment (in hundredths)	− 0.04	(− 0.07, − 0.02)	< .01
Competitive food healthfulness scale score	− 0.02	(− 0.01, 0.07)	.65
NSLP healthfulness scale score	0.03	(− 0.14, 0.20)	.71
Proportion of students eligible for FRPM	0.21	(0.07, 0.34)	< .01
Breakfast served in classroom (ref: No)			
Yes	0.04	(− 0.02, 0.10)	.15

*FRPM* free or reduced-price meal

^a^Generalized linear regression models for panel data with a binomial distribution, probit link function, and robust standard errors

^b^Schools could be observed between 1 and up to 6 school years if they were open during all 6 observed school years. Total school-year observations = 571

**Fig. 1:**
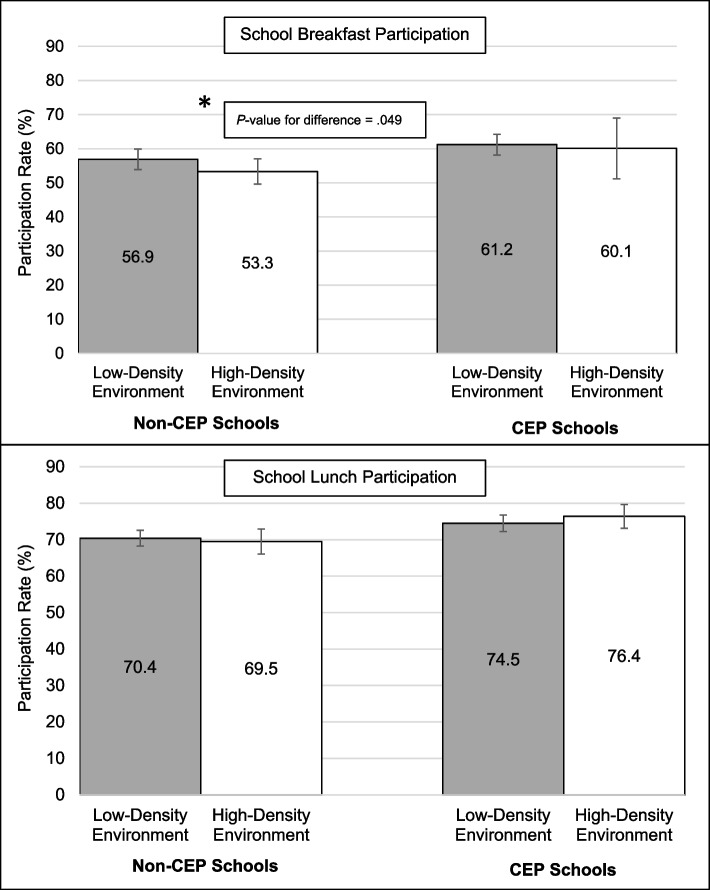
School participation in the Community Eligibility Provision (CEP) as a moderator of the relationship between the community food environment around schools and participation rates in school breakfast and in school lunch. Error bars represent 95% confidence intervals^1^

Subsequent models included an interaction term between the community food environment around schools and school CEP participation to assess whether the relationship between the food environment and school meal participation varies by school CEP participation. Results of regression models including two-way interaction terms are presented in Additional file 1: Tables S1 + S2, and predicted participation rates for school breakfast and school lunch by CEP participation status are shown in Fig. 1.

Based on comparison of predicted participation rates, there was no association between the community food environment and school breakfast participation rates in schools participating in CEP (*P*-value for difference = 0.80), whereas such an association was detected among schools not participating in CEP. Specifically, school breakfast participation rates were significantly lower among non-CEP schools surrounded by a high-density of unhealthy food outlets (predicted participation rate = 53.3%) than among non-CEP schools surrounded by a low-density of unhealthy food outlets (predicted participation rate = 56.9%, *P*-value for difference = 0.049). Nonetheless, the interaction term testing whether CEP modified the relationship between the density of the food environment and breakfast participation was not statistically significant (*B* = 0.07, *P* = 0.62).

The association between school lunch participation rates and density of the community food environment did not differ by school CEP participation (*B* = 0.09, *P* = 0.18). There were no significant relationships between the community food environment and school lunch participation rates for CEP schools nor for non-CEP schools.

**Fig. 2:**
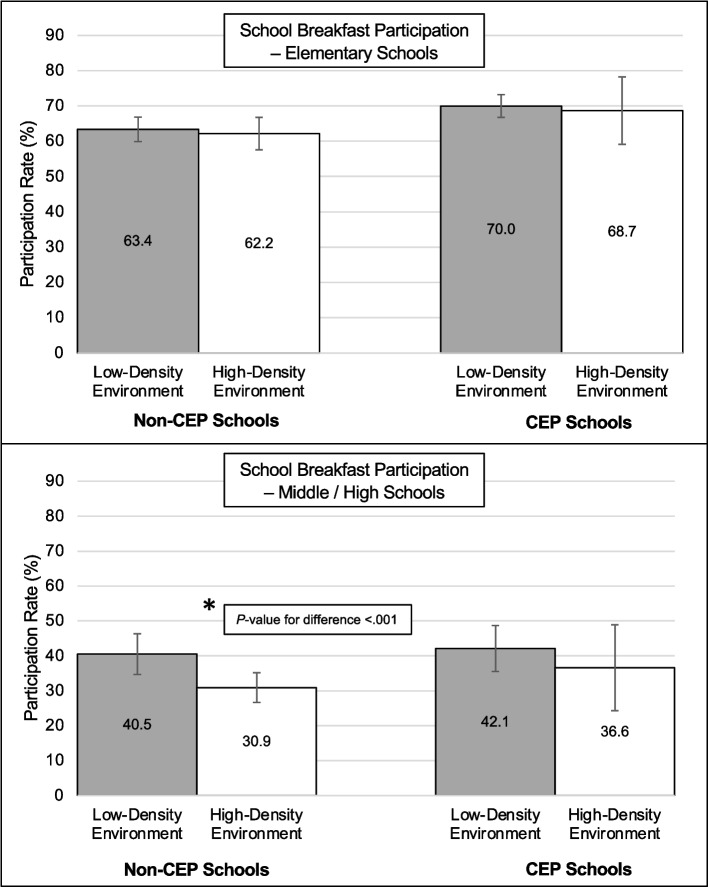
Results from a three-way interaction between school participation in the Community Eligibility Provision (CEP), the community food environment around schools, and school level for participation rates in school breakfast. Error bars represent 95% confidence intervals^1^

Models including 3-way interaction terms between the community food environment around schools, school CEP participation, and school level assessed whether relationships displayed in Fig. 1 differed by school level. Results from regression models with 3-way interaction terms are presented in Additional file 1: Tables S3 + S4, and predicted participation rates for school breakfast by CEP participation status and school level are shown in Fig. 2. Estimates from the model with school breakfast participation rate as the outcome showed that differences in school breakfast participation by the healthfulness of the community food environment that were observed among non-CEP schools were mostly attributable to middle/high schools. Among non-CEP middle/high schools, school breakfast participation rates were significantly lower for schools surrounded by a high-density of unhealthy food outlets (predicted participation rate = 30.9%) than for schools surrounded by a low-density of unhealthy food outlets (predicted participation rate = 40.5%, *P*-value for difference < 0.001), whereas school breakfast participation rates among elementary schools surrounded by a high-density and a low-density food environment were not significantly different from one another (*P*-value for difference = 0.60). Associations between school lunch participation rates and density of unhealthy food outlets did not differ by school CEP status among elementary or middle/high schools; predicted participation rates for school lunch by CEP participation status and school level are shown in Additional file 2: Fig. S1.

All estimates from sensitivity analyses including those treating time as a series of dummy variables and those using 3-level mixed-effects linear regression models produced similar patterns of results (data not shown). Using the 3-level mixed-effects model, school breakfast participation rates were similarly significantly lower among schools not participating in CEP and surrounded by a high-density food environment than among non-CEP schools surrounded by a low-density food environment (predicted difference in participation rate = 3.9%, *P*-value for difference = 0.01). As in main presented results, no significant difference in participation in school breakfast among non-CEP schools was detected (*P*-value for difference = 0.85).

## Discussion

In this study, we examined whether associations between the density of unhealthy food outlets around schools and participation rates in school breakfast and in school lunch differ by school CEP participation, using longitudinal data collected over a 6-year period following the implementation of the policy authorization of CEP. While there was no observed moderation effect of CEP participation on the association between the community food environment around schools and participation in school meals, findings from models including interaction terms between school CEP participation and the community food environment around schools showed that among schools not participating in CEP, predicted school breakfast participation rates were significantly lower when there was a high density of unhealthy food outlets surrounding schools. In contrast, there were no differences based on the community food environment among schools participating in CEP. Models including 3-way interaction terms indicated that the observed differences in school breakfast participation rates by the community food environment around schools among non-CEP schools were mostly attributable to middle/high schools.

Study findings with respect to school breakfast participation build on previous studies showing that increased density of food outlets nearby schools is associated with decreased participation in school meals [14, 15] and that CEP participation is associated with higher school meal participation rates [3, 20]. Results from the current study did not find an overall association between the density of food outlets surrounding schools and school meal participation, as was observed in previous literature (Mirtcheva and Powell) [14]. It is worth noting that the Mirtcheva and Powell study relied upon data collected prior to the authorization of CEP and that in our data we observed an association between the food environment and meal participation in non-CEP schools. Our results, which show that school participation in CEP reduces the influence of unhealthy food outlets surrounding schools on school breakfast participation rates, suggest that CEP participation may have benefits for student health by reducing the purchasing of foods from nearby outlets. Importantly, the food outlets used to characterize the community food environment around schools in this study (limited-service (i.e., fast-food) restaurants and convenience stores) largely sell unhealthy food items [39, 40]. Previous research has also shown that a higher density of food outlets surrounding schools is associated with less healthy food intake [41, 42] and higher BMI among students [43]. In regard to health equity, students from disadvantaged households are more likely to attend schools with higher densities of surrounding unhealthy food outlets [11, 44–46]. For instance, D’Angelo and colleagues showed that low-income and Hispanic students are more likely to attend schools surrounded by fast-food outlets [45] and Zenk and Powell found that schools within lower income neighborhoods were surrounded by comparatively more fast-food restaurants and convenience stores [46].

Regression models did not detect similar differences in associations between the community food environment and participation rates in school lunch according to school CEP participation. This may occur because all schools within the analytical sample have closed campus policies during lunch, and students are not allowed to leave the school during lunch periods. Because of these policies, participation in school lunch may be less influenced by the external community food environment around schools, whereas breakfast participation may face more competition from surrounding food outlets. Future studies capturing schools’ open campus lunch policies would provide additional insights into potential influences of surrounding community food environments on school lunch participation.

A primary strength of our study is that all schools within the sample were predominantly low-income, with high rates of free and reduced-price meal eligibility. As such, we are able to assess potential benefits of CEP participation by comparing participating schools with non-participating but likely eligible schools. Evidence of the benefits associated with CEP participation are consequential because large numbers of CEP-eligible schools do not participate in CEP; for example, 48% of eligible schools in New Jersey did not participate in CEP in SY 2020–2021 [31]. Policies that encourage CEP participation among eligible schools, such as providing higher meal reimbursement rates to schools and simplifying qualification processes, may increase the reach of the program and its associated benefits for students [47–49].

Findings also suggested that the presence of food outlets near schools more strongly influence school breakfast participation among older children and adolescents compared to younger children. We speculate that this may occur because older children and adolescents have comparatively higher levels of autonomy and access to funds to purchase food from nearby outlets than younger children [13]. Consideration of policy actions to expand the reach of CEP may be therefore particularly warranted for middle and high schools.

### Limitations

There are some study limitations that should be noted. First, analyses were based on four school districts, and because CEP participation decisions are often made at the district level, there may be limited variability in CEP participation across schools within a district. To address this, we conducted sensitivity analyses that adjusted for nesting of schools within school districts and these produced similar findings. Second, middle and high schools were combined into a single category because of the limited sample size. Third, because data were collected at the school level, we cannot assess the contribution of food outlets surrounding children’s homes, which may differ in healthfulness from those in close proximity to their schools. However, a previous study conducted within the same school districts indicated that most students live very close to the schools they attend, with 28% of students living within 0.25 miles of the school they attended, and another 29% of students living between 0.25 and 0.5 miles of the school they attended [35]. Fourth, while the schools in our sample were low-income, with high rates of eligibility for free and reduced-price meals, we were not able to obtain the identified student percentages for non-CEP participating schools for earlier years in the sample. However, based on the 2019–2020 data, all schools in the sample in that year had identified student percentages that would make them eligible for CEP. Finally, the majority of schools within the sample served a large proportion of racial/ethnic minority students and were located within urban settings. Consequently, findings may not be generalizable to all students attending schools within the US or abroad.

## Conclusions

This study provides compelling evidence that schools’ CEP participation may lessen the detrimental influence of unhealthy food environments on school breakfast participation rates—particularly among middle/high schools, which enroll older children and adolescents. Findings provide evidence for the benefits of the CEP program and provision of free school meals to children and suggest that policy actions to increase the reach of the program may benefit students. The benefits of policy actions to increase the reach of CEP may be heightened among schools serving older children and adolescents.

### Supplementary Information

Additional file 1: Tables S1-S4. Table S1 – [School breakfast participation by community food environment and CEP participation], Table S2 – [School lunch participation by community food environment and CEP participation], Table S3 – [School breakfast participation by community food environment, CEP participation, and school level], Table S4 – [School lunch participation by community food environment, CEP participation, and school level].

Additional file 2: Fig. S1. Predicted school lunch participation rates by community food environment, CEP participation, and school level. 

## Data Availability

The datasets generated and/or analyzed during the current study are not publicly available because the data are currently being utilized for additional analyses and ongoing research, but are available from the corresponding author on reasonable request.

## Abbreviations

CEP: Community Eligibility Provision
FRPM: Free or reduced-price meals
SY: School year
