# Supplementary material for: The relationship between community food environment around schools and student meal participation: the role of school CEP participation status
Source: BMC Med. 2024 Jul 9;22:287. doi: 10.1186/s12916-024-03498-6 (PMC11232155; doi:10.1186/s12916-024-03498-6)
Supplement: Supplementary file 1 — Additional file 1: Tables S1-S4. Table S1 – [School breakfast participation by community food environment and CEP participation], Table S2 – [School lunch participation by community food environment and CEP participation], Table S3 – [School breakfast participation by community food environment, CEP participation, and school level], Table S4 – [School lunch participation by community food environment, CEP participation, and school level]. [file 12916_2024_3498_MOESM1_ESM.docx]

**ADDITIONAL FILE 1**

**Table S1.** Results from a regression model^1^ assessing predictors of average daily participation (ADP) rates in school breakfast, including an interaction effect for the community food environment around schools among 126 schools observed over a 6-year period.^2,3^

| Predictor Variable | Coef. | 95% CI | *P*-value |
| --- | --- | --- | --- |
| School breakfast |  |  |  |
| Community food environment around school (ref: Low-density) |  |  |  |
| High-density | -0.10 | (-0.20, -0.001) | .049 |
| Participation in CEP (ref: No) |  |  |  |
| Yes | 0.12 | (0.02, 0.22) | .03 |
| Community food environment around school * Participation in CEP | 0.07 | (-0.20, 0.33) | .62 |

^1^ Generalized linear regression models for panel data with a binomial distribution, probit link function, and robust standard errors

^2^ Schools could be observed between 1 and up to 6 school years if they were open during all 6 observed school years. Total school-year observations = 571.

^3^ Models adjusted for school year and school-level variables including majority race/ethnicity (>50% black, >50% Hispanic, >50% non-Hispanic White / no majority race/ethnicity), enrollment, school level (elementary school vs. middle/high school), competitive food healthfulness (scale ranging between 0-1), NSLP healthfulness (scale ranging between 0-1), proportion of students eligible for free or reduced-price meals, and breakfast model (breakfast served in classroom vs. not).

**Table S2.** Results from a regression model^1^ assessing predictors of average daily participation (ADP) rates in school lunch, including an interaction effect for the community food environment around schools among 126 schools observed over a 6-year period.^2,3^

| Predictor Variable | Coef. | 95% CI | *P*-value |
| --- | --- | --- | --- |
| School lunch |  |  |  |
| Community food environment around school (ref: Low-density) |  |  |  |
| High-density | -0.03 | (-0.12, 0.07) | .57 |
| Participation in CEP (ref: No) |  |  |  |
| Yes | 0.13 | (0.07, 0.19) | <.001 |
| Community food environment around school * Participation in CEP | 0.09 | (-0.04, 0.22) | .18 |

^1^ Generalized linear regression models for panel data with a binomial distribution, probit link function, and robust standard errors

^2^ Schools could be observed between 1 and up to 6 school years if they were open during all 6 observed school years. Total school-year observations = 571.

^3^ Models adjusted for school year and school-level variables including majority race/ethnicity (>50% black, >50% Hispanic, >50% non-Hispanic White / no majority race/ethnicity), enrollment, school level (elementary school vs. middle/high school), competitive food healthfulness (scale ranging between 0-1), NSLP healthfulness (scale ranging between 0-1), proportion of students eligible for free or reduced-price meals, and breakfast model (breakfast served in classroom vs. not).

**Table S3.** Results from a regression model^1^ assessing predictors of average daily participation (ADP) rates in school breakfast, including a three-way interaction between school participation in the Community Eligibility Provision (CEP), the community food environment around schools, and school level among 126 schools observed over a 6-year period.^2,3^

| Predictor Variable | Coef. | 95% CI | *P*-value |
| --- | --- | --- | --- |
| School breakfast |  |  |  |
| Community food environment around school (ref: Low-density) |  |  |  |
| High-density | -0.03 | (-0.15, 0.09) | .60 |
| Participation in CEP (ref: No) |  |  |  |
| Yes | 0.18 | (0.06, 0.30) | .003 |
| School level (ref: Elementary school) |  |  |  |
| Middle or high school | -0.59 | (-0.77, -0.41) | <.001 |
| Community food environment around school * Participation in CEP | -0.003 | (-0.29, 0.28) | .98 |
| School level * Participation in CEP | -0.14 | (-0.32, 0.04) | .14 |
| Community food environment around school * School level | -0.23 | (-0.39, -0.07) | .005 |
| Community food environment around school * Participation in CEP * School level | 0.12 | (-0.32, 0.55) | .59 |

^1^ Generalized linear regression models for panel data with a binomial distribution, probit link function, and robust standard errors

^2^ Schools could be observed between 1 and up to 6 school years if they were open during all 6 observed school years. Total school-year observations = 571.

^3^ Models adjusted for school year and school-level variables including majority race/ethnicity (>50% black, >50% Hispanic, >50% non-Hispanic White / no majority race/ethnicity), enrollment, competitive food healthfulness (scale ranging between 0-1), NSLP healthfulness (scale ranging between 0-1), proportion of students eligible for free or reduced-price meals, and breakfast model (breakfast served in classroom vs. not).

**Table S4.** Results from a regression model^1^ assessing predictors of average daily participation (ADP) rates in school lunch, including a three-way interaction between school participation in the Community Eligibility Provision (CEP), the community food environment around schools, and school level among 126 schools observed over a 6-year period.^2,3^

| Predictor Variable | Coef. | 95% CI | *P*-value |
| --- | --- | --- | --- |
| School lunch |  |  |  |
| Community food environment around school (ref: Low-density) |  |  |  |
| High-density | -0.07 | (-0.16, 0.02) | .13 |
| Participation in CEP (ref: No) |  |  |  |
| Yes | 0.18 | (0.12, 0.24) | <.001 |
| School level (ref: Elementary school) |  |  |  |
| Middle or high school | -0.43 | (-0.58, -0.27) | <.001 |
| Community food environment around school * Participation in CEP | 0.12 | (-0.01, 0.25) | .08 |
| School level * Participation in CEP | -0.09 | (-0.20, 0.03) | .15 |
| Community food environment around school * School level | 0.11 | (-0.01, 0.32) | .30 |
| Community food environment around school * Participation in CEP * School level | -0.05 | (-0.49, 0.38) | .81 |

^1^ Generalized linear regression models for panel data with a binomial distribution, probit link function, and robust standard errors

^2^ Schools could be observed between 1 and up to 6 school years if they were open during all 6 observed school years. Total school-year observations = 571.

^3^ Models adjusted for school year and school-level variables including majority race/ethnicity (>50% black, >50% Hispanic, >50% non-Hispanic White / no majority race/ethnicity), enrollment, competitive food healthfulness (scale ranging between 0-1), NSLP healthfulness (scale ranging between 0-1), proportion of students eligible for free or reduced-price meals, and breakfast model (breakfast served in classroom vs. not).
