# Supplementary material for: The relationship between community food environment around schools and student meal participation: the role of school CEP participation status
Source: BMC Med. 2024 Jul 9;22:287. doi: 10.1186/s12916-024-03498-6 (PMC11232155; doi:10.1186/s12916-024-03498-6)
Supplement: Supplementary file 2 — Additional file 2: Fig. S1. Predicted school lunch participation rates by community food environment, CEP participation, and school level. [file 12916_2024_3498_MOESM2_ESM.docx]

**Fig. S1.** Results from a three-way interaction between school participation in the Community Eligibility Provision (CEP), the community food environment around schools, and school level for participation rates in school lunch. Error bars represent 95% confidence intervals.^1^

School Lunch Participation –

Middle / High Schools

School Lunch Participation – Elementary Schools

^1^ Participation rates predicted using margins command in Stata, based on coefficients from regression models (Table 1 in Supplementary Materials) including an interaction term between the community food environment (low vs. high density of unhealthy food outlets) and school CEP participation status (yes vs. no)
